# Genome- and Toxicology-Based Safety Assessment of Probiotic *Akkermansia muciniphila* ONE Isolated from Humans

**DOI:** 10.3390/foods13131979

**Published:** 2024-06-24

**Authors:** Na Lv, Caiping Wang, Hongtao Zhou, Xin Ma, Xueping Yu, Dayong Ren

**Affiliations:** 1 College of Food Science and Engineering, Jilin Agricultural University, Changchun 130118, China; lvna19802003@126.com (N.L.); wang_caiping2019@163.com (C.W.); zht981022@outlook.com (H.Z.); 2 State Key Laboratory of Bioreactor Engineering, East China University of Science and Technology, Shanghai 200237, China; pkartest@yeah.net (X.M.); yuxueping@thankcome.com (X.Y.)

**Keywords:** *A. muciniphila ONE*, genome sequencing, genome annotation, safety assessment, probiotic

## Abstract

In this study, the genome of *Akkermansia muciniphila* ONE (designated AKK ONE) was sequenced, assembled, and analyzed. In addition, the safety of this strain was further evaluated by toxicological studies. The results showed that the AKK ONE genome is contained on a single chromosome with a total length of 2,817,524 bp and an average GC content of 55.48%. In total, 2411, 1131, 1168, 1745, and 1402 genes were annotated to the NR, GO, KEGG, COG, and SwissProt database, respectively. Potential resistance genes, adeF, tetW, ANT(3″)-IIa, and aadA1 were detected. AKK ONE was sensitive to ampicillin, ceftriaxone, cefotaxime, meropenem, tetracycline, and chloramphenicol and resistant to moxifloxacin. No potential virulence-related genes were detected. The PathogenFinder database analysis showed that AKK ONE was a non-potential human pathogen. This strain had good gastroenteric fluid tolerance and a weak ability to colonize the gut. No test item-related adverse effects were observed in the acute and subchronic toxicity test. AKK ONE did not display mutagenic activity either. This strain did not change the hematological and clinical biochemical parameters of mice. The weights of the organs were not affected by AKK ONE treatment. These results support that AKK ONE is safe for use as a probiotic at a dose of 8.28 × 10^9^ CFU/kg bw/day.

## 1. Introduction

Probiotics are living microorganisms that are beneficial to the health of the host. The primary requirement for a newly isolated strain to be a probiotic is that it is safe at the intended levels of use in food [1]. At present, research in the field of probiotics has mainly focused on traditional bacterial species, such as those belonging to the genera *Lactiplantibacillus* and *Bifidobacterium*. In recent years, it has been found that some symbiotic bacteria present in the human gastrointestinal tract also have probiotic properties and exhibit a superior effect compared with plant- and dairy-based bacteria [2].

*Akkermansia muciniphila* (*A. muciniphila*) is a Gram-negative anaerobic bacterium present in the feces of healthy adults that colonizes the gut within 1 year of birth. In general, it accounts for 1–4% of the bacteria in the feces of healthy adults [3]. The mucosal layer of the gastrointestinal tract is the site of *A. muciniphila* colonization [4], and its numbers usually decrease with age [5]. However, interestingly, rich levels of *A. muciniphila* were detected in the gastrointestinal tract of several centenarians. Colonization of this bacterium can effectively increase mucus thickness and improve intestinal barrier function [6], thus inducing a series of metabolic and immune responses in the host [7]. Recently, it has received a lot of attention because increased levels of *A. muciniphila* in the gastrointestinal tract have been linked to the prevention of obesity, aging, and diabetes [8,9]. In 2022, the key role of this bacterium in the treatment of malignancies was discovered [10]. It is worth noting that oral supplementation of *A. muciniphila* can reduce the serum triglyceride level of the host and increase the insulin sensitivity of the cells [11]. Some studies have shown that the absence or reduction of *this* bacterium is associated with the occurrence of a variety of diseases [12,13]. After taking *A. muciniphila*, a partial reduction in symptoms associated with these diseases was observed [11,14], thus indicating its potential as a next-generation probiotic. In recent years, *A. muciniphila* has received a lot of attention as a promising next-generation probiotic [9,15]. However, some reports suggest that the beneficial effects of this bacterium are strain dependent. For example, different strains of *A. muciniphila* exhibited different effects on inflammatory bowel disease and colitis [16,17].

Although *A. muciniphila* has some biological activities, current studies did not provide sufficient evidence to support the safety of it for use as a probiotic. At present, the data on the safety evaluation of this bacterium are still very limited. In order to promote *A. muciniphila* application in the probiotic industry, it is urgent to evaluate and confirm its safety. To date, only a few strains, such as BAA-835, DSM 22959, and AM06, have been evaluated for safety [18,19,20]. Therefore, the safety of new strains at the strain level needs to be considered and evaluated before clinical application.

The genome is the carrier of the genetic information of the organism. The determination and analysis of the whole genome sequence of the strain can accurately locate the excellent genes and interpret their physiological regulation mechanism from the molecular level [21]. It also provides the basis for deep exploration of its metabolism. In addition, the in-depth study of gene structure characteristics can provide a theoretical basis for the further application and improvement of these excellent genes by means of genetic engineering [22,23]. The genome-based microbiological safety assessment is becoming more and more popular because of its fast and comprehensive characteristics [24,25]. Through the study of genome sequence, the potential pathogenic genes can be found. Therefore, targeted regulation can be carried out to make a more reasonable and safe use of microbial resources. Whole genome sequencing (WGS) provides more information on the genome sequence characteristics and gene function of the strains. Through a comparison with different annotation databases, the genes related to the survival and metabolism of the strain can be preliminarily identified, which provides a basis for the further utilization of the strain.

However, safety assessments based on whole genome sequences are not very reliable. Therefore, it is necessary to comprehensively determine the safety of *A. muciniphila* combined with the results of systematic toxicological evaluation. We isolated a new strain of *A. muciniphila* from human feces. In accordance with FAO/WHO recommendations, phenotypic testing, taxonomic identification, genome sequence characteristics analysis, presumed virulence, and antibiotic-resistance gene identification analyses, combined with in vivo toxicity testing and mutagenicity analysis, were performed to assess the potential of the strains isolated in our study as probiotics.

## 2. Materials and Methods

### 2.1. Bacterial Strains and 16S rRNA Gene Sequencing

The bacterial strains of *A. muciniphila ONE* (designated *AKK ONE*) used in this study were previously isolated from the feces of healthy adults. This strain is stored in the China General Microbial Culture Collection Center (Beijing, China), with the preservation number CGMCC No. 20954. Brain Heart Infusion (BHI) broth medium (BD Difco, San Jose, CA, USA) was used to culture AKK ONE. The 16S rRNA gene sequencing of the isolated strain AKK ONE was completed by Thankcome Biotechnology Co., Ltd. (Suzhou, China). MEGA software v11.7 was used to display the 16S rDNA sequence phylogenetic tree of AKK ONE and related species by the adjacent linking method. The similarity calculation was performed 1000 times.

### 2.2. Genome Sequencing, Assembly, and Analysis

PacBio Sequel II platform (Frasergen Bioinformatics Co., Ltd., Wuhan, China) was used for genomic DNA extraction, DNA quality and quantity determination, and DNA fragmentation and sequencing. The raw sequencing reads generated from the PacBio platforms were processed by SMRTlink v8.0. As part of the Canu (version 2.0) assembly process, the error correction of the reads was performed. The genome was assembled using Canu v2.0. When the best overlap diagram was circular, Canu v2.0 detected and labeled the final assembly as circular. As an alternative approach, we confirmed genome circularization by using BLASTN (version 2.7.1) to identify repeated sequences at both ends. The NCBI Prokaryotic Genome Automatic Annotation Pipeline (PGAP) was used to complete the genome annotation. The genes were annotated by comparing them with genes held in non-redundant protein (NR), Cluster of Orthologous Groups of proteins (COG), Gene Ontology (GO), Kyoto Encyclopedia of Genes and Genomes (KEGG) and carbohydrate-active enzymes (CAZymes) database.

### 2.3. Analysis of Antibiotic-Resistance Gene, Virulence Gene, and Pathogenicity of AKK ONE

Two databases, the CARD (The Comprehensive Antibiotic Resistance Database, https://card.mcmaster.ca/, accessed on 5 August 2023) and ResFinder (https://cge.cbs.dtu.dk/services/ResFinder/, accessed on 17 September 2023), were used to predict potential resistance-related genes of AKK ONE. Diamond (v0.9.12.113) software was used to compare the protein sequence of AKK ONE gene with the CARD database. In addition, the sensitivity of AKK ONE to ampicillin, ceftriaxone, cefotaxime, meropenem, tetracycline, moxifloxacin, and chloramphenicol was determined using the AGAR dilution method recommended by the Clinical and Laboratory Standards Institute (CLSI). The quality-control strain, Bacteroides fragilis ATCC25285, was determined using the microbroth dilution method recommended by CLSI. Bacterial virulence factors were analyzed in the VFDB database (http://www.mgc.ac.cn/VFs/main.htm, accessed on 29 August 2023). Sequence similarity and sequence coverage greater than 70% were used as screening criteria. In addition, virulence related genes were compared using the VirulenceFinder database (https://cge.cbs.dtu.dk/services/VirulenceFinder, accessed on 26 September 2023). Sequence similarity greater than 90% and sequence coverage greater than 60% were used as screening criteria. The PathogenFinder database was used to predict the pathogenicity of AKK ONE by analyzing the protein sequence, genome sequence, or original sequence of the strain.

### 2.4. Gastrointestinal Tract (GI) Tolerance, Hydrophobicity, Auto-Aggregation, and Biofilm Formation Ability

#### 2.4.1. Tolerance to Artificial Gastric and Intestinal Fluid

Gastroenteric fluid tolerance was measured using the method described by Ulsemer et al. [26]. The modified simulated gastric fluid (pH 2.0) was composed of 2 g/L NaCl, 5 M HCl and 3.2 g/L pepsin. The simulated intestinal fluid (pH 8.0) was prepared with 6.8 g/L KH_2_PO_4_, 10 g/L pancreatic enzyme, and 0.1 M NaOH. AKK ONE cultured overnight was centrifuged at 4000 rpm at 4 °C for 10 min, washed, re-suspended in physiological saline (control) or gastric/intestinal fluid, and incubated at 37 °C. The number of viable bacteria was measured at 0 h and 3 h, respectively. The survival rate of AKK ONE in artificial gastric or intestinal fluid was calculated by the ratio of 3-h viable count to 1-h viable count.

#### 2.4.2. Hydrophobicity

According to the method described by Cozzolino et al. [19], the hydrophobicity of AKK ONE was performed using bacterial adhesion test to hydrocarbons (BATH), with some modifications. AKK ONE was cultured overnight in BHI medium at 37 °C. Cells were collected by centrifugation at 8000 rpm, 4 °C, for 10 min. Then, cell precipitates were washed twice and re-suspended in a phosphate buffer (pH 7.0). The absorbance of the solution at the wavelength of 600 nm was adjusted to 0.5 to standardize the count of bacteria. Then, an equal volume of dimethyl benzene was added and mixed. After incubation at room temperature for 15, 30, and 60 min, the water phase was carefully extracted, and its absorbance at 600 nm was measured. The hydrophobicity of AKK ONE was calculated using the following formula:

(1) $$Hydrophobicity\;\%=\frac{p0-p1}{P1}\times 100$$

where *p*0 and *p*1 are the absorbance values of the solution before and after hydrocarbon extraction, respectively.

#### 2.4.3. Auto-Aggregation

Automatic aggregation measurement was performed as described by Cozzolino et al. The bacterial suspension was cultured at 37 °C, and its absorbance at 600 nm was measured at 4, 8, 12, 16, 20, and 24 h. We used the following formula to calculate the percentage of self-aggregation:

(2) $$Auto-aggregation\;\%=1-\frac{p1}{P0}\times 100$$

where *P*0 is the absorbance at time 0; and *p*1 is the absorbance detected after 4, 8, 12, 16, 20, and 24 h.

#### 2.4.4. Biofilm Formation Ability

According to the methods of Dey et al. [27], the biofilm-forming ability of strain AKK ONE was analyzed. AKK ONE was cultured overnight in tryptone soy broth (TSB) (BD Difco, USA) at 37 °C and harvested by centrifugation at 8000 rpm for 10 min at 4 °C. The bacterial cells were washed twice with PBS and re-suspended in TSB medium without glucose and in TSB medium with 0.25%, 1%, and 2.5% glucose. Then, 200 μL of each bacterial suspension was loaded into a 96-well plate. Uninoculated medium was used as negative control. Plates were cultured at 37 °C for 24 h. The cells were then immobilized with 200 μL methanol and stained with 200 μL of 2% crystal violet for 5 min. The adherent cells were suspended in 160 μL of 33% (*v*/*v*) glacial acetic acid. Their absorbance at 600 nm was measured. The critical value (OD_0_) is defined as the average OD value of the negative control. According to OD values, the strains were classified as non-biofilm producers (OD ≤ OD_0_), weak biofilm producers (OD_0_ ≤ OD ≤ 2 × OD_0_), medium biofilm producers (2 × OD_0_ ≤ OD ≤ 4 × OD_0_), or strong biofilm producers (4 × OD0 ≤ OD).

### 2.5. Acute Toxicity

To evaluate the acute toxicity of AKK ONE, SPF-grade Kunming mice were randomly and equally divided into four groups. Each group was administrated with AKK ONE suspension containing 1 × 10^9^, 5 × 10^10^, and 5 × 10^11^ colony-forming units (CFU)/day, or saline (0.5 mL/day). Changes in animal behavior and body weight were observed and recorded daily. The experiment was carried out continuously for 7 days. At the end of the experiment, blood samples were collected for hematological and serum biochemical analyses. Histopathological examination of major organs and tissues was performed on all animals in each group. The protocol was approved by the Ethics Committee of Laboratory Animal Center of Jilin Agricultural University (Permit Number: 20220711002).

### 2.6. In Vitro Mammalian Cell Micronucleus Test

In vitro micronucleus test of mammalian cells was carried out with reference to Fenech’s [28] cytodynamic retardation method. In total, 40 Kunming mice (20 male and 20 female), 7 weeks old, were selected for this experiment, with 5 females and 5 males in each group. Mice in the three experimental groups were given a bacterial suspension of 222.2, 666.7, and 2000 mg/kg body weight via intragastriction, respectively. The negative control was intragastric with distilled water. The positive control group was given a one-time injection of cyclophosphamide (dissolved in sterile normal saline) at 60 mg/kg body weight. After euthanasia, bone marrow samples were collected immediately from each mouse’s femur for smears and staining. The micronucleus rate of each mouse was measured separately.

### 2.7. Bacterial Reverse Mutation Test (Ames Test)

Bacterial regression mutation test was performed using *Salmonella typhimurium* (*S. typhimurium*) TA97a, TA98, TA100, and TA1535 and *Escherichia coli* (*E. coli*) WP2 uvrA. AKK ONE was dissolved in sterile distilled water and centrifuged for 10 min to remove residual bacteria. The solution was then filtered by a 0.22 μm filter membrane. The concentration of the solution reached the maximum recommended concentration of 5000 μg/plate in the OECD test guidelines. Dixon, sodium azide, methyl methylsulfonate, and 2-aminofluorene were used as positive controls. The survival rate of bacterial cells was examined by the plate test. If there was a dose-related increase in the number of responders, the subject was considered mutagenic. The increase was considered biologically relevant if the number of reversals in *S. typhimurium* TA98 and TA100 strains and the *E. coli* WP2 uvrA strain was more than twice as high as that of the negative control. In the *S. typhimurium* TA97a and A1535 strains, it was considered biologically relevant when the number of reversals was more than three times that of the negative control.

### 2.8. Ninety-Day Oral Toxicity Study

The oral subchronic toxicity of AKK ONE was evaluated according to the OECD testing guidelines. In total, 64 Kunming mice (32 male and 32 female) with a body weight of 18–22 g were randomly divided into 4 groups, with 4 females and 4 males in each group. Group 1 was given saline daily. The remaining three experimental groups were given 9.2 × 10^8^, 2.76 × 10^9^, and 8.28 × 10^9^ CFU/kg body weight per day, respectively. Normal saline and bacterial suspensions were administered by gavage for 90 consecutive days. Clinical signs were recorded daily. The changes in body weight, food consumption, and blood biochemical indexes were recorded every day. At the end of the experiment, a gross histopathological examination was performed.

### 2.9. Statistics

All experiments were carried out in triplicate. Data were presented as means ±standard deviation (SD). Data were statistically analyzed by analysis of variance (ANOVA), with SPSS statistical software v. 26.0.0. Significant differences were performed by Tukey’s multiple comparison. A probability of *p <* 0.05 was considered statistically significant. Graph Pad Prism V 8.0 software was used for the analysis.

## 3. Results and Discussion

### 3.1. Microbiological Properties and 16S rRNA Gene Sequencing

**Figure 1:**
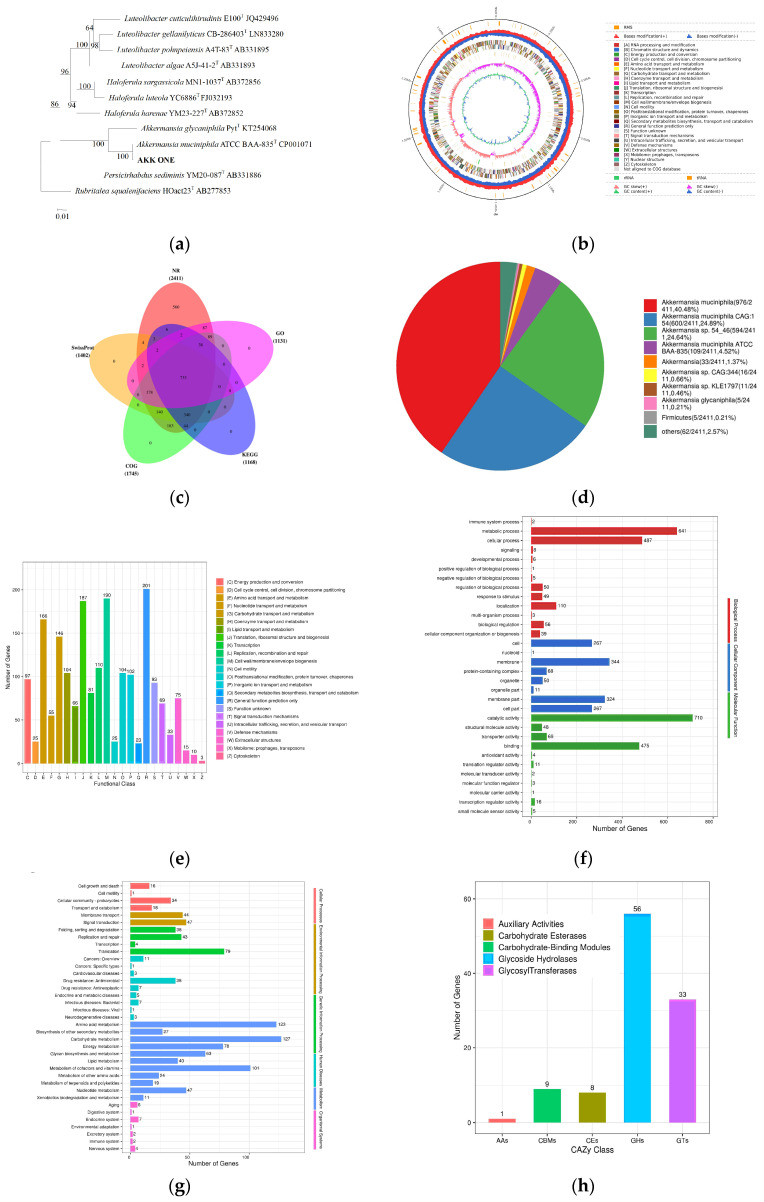
Genome sequence characteristics and functional annotation. (**a**) Phylogenetic representation of AKK ONE. The scale bar represents 0.01 substitutions per nucleotide position. Only the value of Bootstrap greater than 50% is displayed on the phylogenetic tree node. The “T” above indicates the type of strain. (**b**) Circular representation of *A. muciniphila ONE* genome. (**c**) NR, COG, GO, SwissProt, and KEGG database annotation statistics. (**d**) Top 10 species distribution chart of NR database. (**e**) COG classification statistics. (**f**) GO classification statistics. (**g**) KEGG classification statistics. (**h**) CAZymes classification of protein functions.

After the anaerobic culture of AKK ONE at 37 °C in BHI medium for 7 days, the colony was light yellow, round, moist, opaque, and neat on the surface (Supplementary Figure S1a). The cells were rod-shaped, with a width of 0.6–0.8 μm and a length of 0.8–4.1 μm, arranged single or in pairs, and Gram-negative (Supplementary Figure S1b). The 16S rDNA sequence phylogenetic tree of AKK ONE and related species was displayed by using MEGA software v11.7. *A. muciniphila* is divided into three phylogenetic groups. To date, most current studies have focused on the strain *A. muciniphila* ATCC BAAs-835 [29]. According to this, a sequence comparison showed that AKK ONE was 99.74% similar to BAA-835 (Figure 1a).

### 3.2. Genome Sequence Characteristics and Functional Annotation

The first available *A. muciniphila* genome, ATCC BAA-835, was sequenced in 2011, comprising one circular chromosome of 2.66 Mbp [30]. In this study, the third generation of whole genome sequencing technology PacBio Sequel II was used to provide technical support for the mining of the AKK ONE genome, which is contained on a single chromosome with a total length of 2,817,524 bp and an average GC content of 55.48% (Supplementary Table S1). Figure 1b depicts a gene circle map constructed using combined data from genome sequencing, gene prediction, and non-coding RNA prediction. A statistical chart for analyzing common and specific annotations in the NR, COG, GO, SwissProt, and KEGG databases is shown in Figure 1c. In total, 2411 genes could be annotated to the NR database (Figure 1c), most of which belonged to the genus *A. muciniphila* (Figure 1d). A total of 60.23% of AKK ONE genes were successfully annotated in the COG database (Figure 1e). Moreover, 201 genes were associated with general function prediction; 190 genes were involved in the synthesis of cell wall, membrane, and envelope; and 187 genes might be responsible for translation, ribosomal structure, and biogenesis. The GO classification statistical results are shown in Figure 1f. In the AKK ONE genome, there were 710 and 641 genes involved in catalytic activity and metabolic processes, respectively. After a comparison with the KEGG database, the functional annotations of 1168 protein-coding genes were obtained (Figure 1g), and 127 of these genes are involved in carbohydrate metabolism. There are 123 genes associated with amino acid metabolism, and 107 genes were annotated in the CAZymes database (Figure 1h). The number of genes encoding glycoside hydrolases (GHs) was the highest, followed by glycosyl transferases (GTs), carbohydrate binding modules (CBMs), carbohydrate esterases (CEs), and auxiliary active enzymes (AAs).

Our research identified genes encoding the cell function, components synthesis, catalytic activity, and metabolic processes by comparing the NR, COG, GO, SwissProt, and KEGG databases. However, the function of 16.78%, 51.61%, 39.77%, 59.68%, and 60.96% of the genes was not annotated in the NR, SwissProt, COG, KEGG, and GO database (Supplementary Table S2), respectively, indicating that AKK contains a large number of genes with unknown functions.

### 3.3. Analysis Results of Antibiotic-Resistance Gene, Virulence Gene, and Pathogenicity of AKK ONE

Antibiotic resistance is a noteworthy issue in the evaluation of probiotic candidates. Potential resistance genes related to adeF, tetW, and ANT(3″)-IIa were detected using the database CARD 3.1.2 (Supplementary Table S3). These genes were potentially associated with resistance to quinolones, tetracyclines, and aminoglycosides, respectively. The adeF gene is a member of the adeFGH gene cluster. When adeF, adeG, and adeH genes are co-expressed in the gene cluster, multidrug-resistance efferent pump AdeFGH can be encoded to make the strain resistant to fluoroquinolones and other drugs. After the ResFinder database comparison, two potential drug resistance-related genes, tet(W) and aadA1, were detected which were associated with tetracycline and aminoglycoside antibiotic resistance, respectively (Supplementary Table S4). However, the CLSI-recommended AGAR dilution test showed that AKK ONE was sensitive to ampicillin, ceftriaxone, cefotaxime, meropenem, tetracycline, and chloramphenicol and resistant to moxifloxacin (Table 1). Although tetracycline and aminoglycoside antibiotic-resistance genes were detected in AKK ONE by comparison with the CARD and ResFinder databases, the AGAR dilution assay recommended by CLSI did not reveal resistance to either class of antibiotics. However, moxifloxacin antibiotic resistance was detected in this strain, which may be related to the presence of the adeF gene in AKK ONE. The result was consistent with that of A. muciniphila AM06 reported by Hou et al. [20]. Notably, Cozzolino et al. reported that DSM 22959 was resistant to chloramphenicol [19]. Therefore, our results were not exactly the same as those previously reported.

The results of the bacterial virulence factor analysis using the VFDB database showed that one potential virulence-related gene (*tufa*) was detected (Supplementary Table S5). No potential virulence-related genes were detected via the VirulenceFinder database comparison. The PathogenFinder database analysis showed that AKK ONE was a non-potential human pathogen. (Supplementary Table S6). The virulence factor and pathogenicity are the main indicators when considering whether a strain can be used as a probiotic. Although the bacterial virulence factor analysis using the VFDB database detected one potential virulence-related gene (*tufa*), *tufa* encodes the translation extension factor Tu 1, which is involved in the translation process of proteins and is widely found in prokaryotes. In addition, no potential virulence-related gene was detected via the VirulenceFinder database comparison. Moreover, the PathogenFinder database analysis revealed that AKK ONE is a non-potential human pathogen. These results elucidated the safety of AKK ONE from a genomic perspective. To date, *A. muciniphila* has not been found to be associated with any disease [31].

### 3.4. GI Tolerance, Hydrophobicity, Auto-Aggregation, and Biofilm Formation

**Figure 2:**
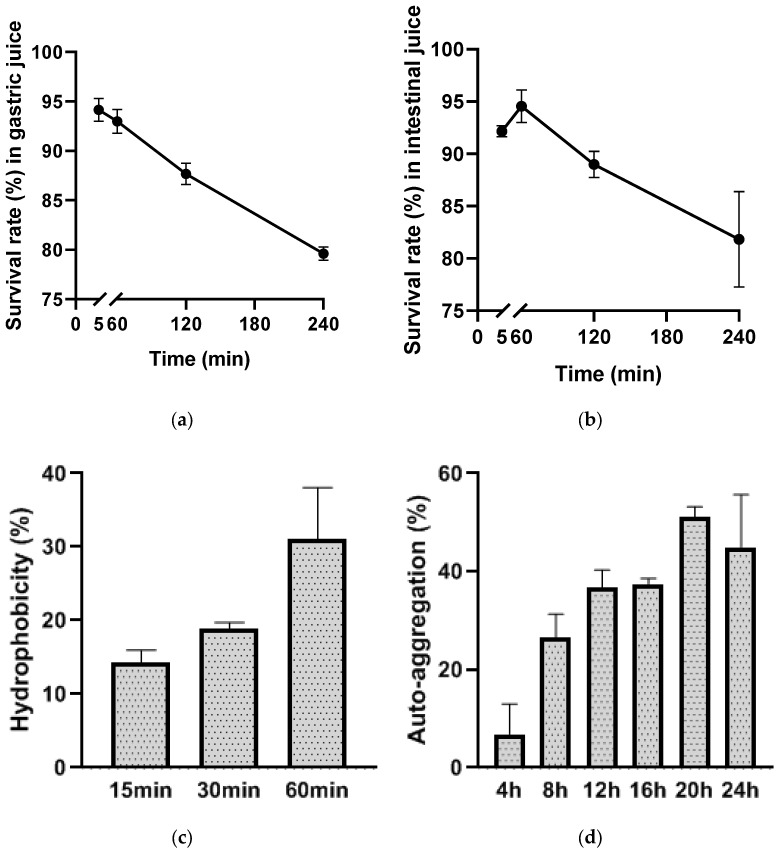
Gastrointestinal tract (GI) tolerance (**a**,**b**), hydrophobicity (**c**) and auto-aggregation (**d**) of AKK ONE. The experiments were repeated three times. Values are means ± SD (*n* = 3).

To determine the tolerance of the strain in the digestive tract, the survival rates of AKK ONE after exposure to artificial gastric and intestinal fluids in vitro were measured. The results showed that the survival rates of AKK ONE in artificial gastric fluid decreased with the time extension (Figure 2a). This strain was basically tolerant to simulated gastric juices and had a survival rate of more than 80% within 3 h compared to the control strain. On the other hand, the survival rate of AKK ONE in simulated intestinal fluid was about 82.3% at 4 h (Figure 2b). These results indicated that AKK ONE has good gastroenteric fluid tolerance. Its tolerance to GI indicates its desirable probiotic properties.

The adhesion of AKK ONE was evaluated by measuring its biofilm formation, hydrophobicity and self-aggregation ability. The formation of biofilms depends on the hydrophobic surface potential of cells. Therefore, in this study, xylene (polar solvent) was used for BATH determination to evaluate the adhesion of bacteria. Figure 2c shows that the hydrophobicity of AKK ONE increased with time, reaching more than 30% at 60 min. As shown in Figure 2d, the auto-aggregation of AKK ONE increased with time and tended to be stable at about 50% at 20 h. After incubating in TSB containing different concentrations of glucose (0%, 0.25%, 1%, and 2.5%) at 37 °C for 24 h, we found that the biofilm formation ability of AKK ONE was weak (Supplementary Table S7). Cozzolino et al. reported that *A. muciniphila* DSM 22959 also showed a lower aptitude in biofilm formation [19]. Combined with these experimental results, it was finally determined that AKK ONE had a weak ability to colonize the gut.

### 3.5. Acute Toxicity

The acute toxicity test is one of the main parts of basic toxicology research. The ultimate purpose of acute toxicity test of exogenous substances in laboratory animals is to clarify the acute toxicity and harm intensity of the substances to human beings. Acute toxicity test can clarify the relative toxicity, mode of action, and special toxicity of a chemical and determine its dose–response relationship, so as to have a preliminary understanding of the toxicity of a chemical, including clinical symptoms; physiological, biochemical, and pathological changes; toxic properties of the subject; and possible target organs. It also provides a basis for the study of the biotransformation process and dynamic changes in the subject’s body. Therefore, in this study, acute toxicity test was used to verify the safety of AKK ONE in vivo to preliminarily evaluate its risk of harm to human body. Because of their strong fecundity, short life span, and similar diet to humans, rodents are often used in the acute toxicity test of substances. Therefore, mice were selected as experimental animals in this study.

**Figure 3:**
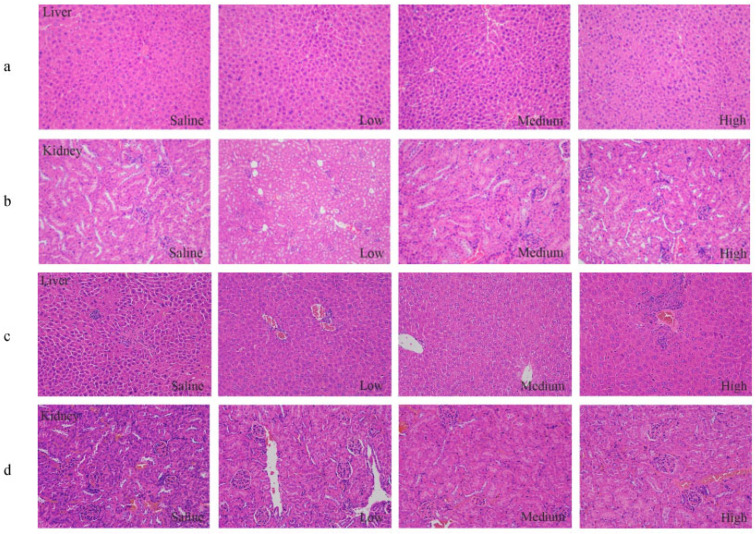
Histopathological analyses of the liver and kidney (H&E, ×200). (**a**,**b**) Pathological sections of the liver and kidney in acute toxicity study, respectively. (**c**,**d**) Pathological sections of the liver and kidney in sub-chronic toxicity study, respectively.

During the experiment, no treatment-related deaths or behavioral abnormalities were observed in the mice. There was no significant difference in body weight and food intake between the AKK ONE treatment group and the control group. No significant difference in hematological values, such as white blood cell count, red blood cell count, hemoglobin, mean corpuscular volume, platelet count, lymphocytes, mean corpuscular hemoglobin concentration, and mean corpuscular hemoglobin, was found between the groups (Table 2). There was no significant difference in blood glucose, triglyceride, cholesterol, bile acid, total protein, alanine aminotransferase, and aspartate aminotransferase levels between the tested mice and the control mice in their serum and liver (Table 3). The creatinine and urea nitrogen in serum did not differ between the groups. There was no significant difference in the weight of the heart, liver, spleen, kidney, thymus, brain, testis, lung, stomach, and intestine between the tested mice and the control (Table 4). In addition, AKK ONE did not change the tissue structure of the liver and kidney (Figure 3a,b).

### 3.6. In Vitro Mammalian Cell Micronucleus Test

As shown in Table 5, there was significant difference in the micronucleus rate between positive control group and negative control group (*p* < 0.001), indicating that the results are reliable. However, no significant difference was found between each dose group and the negative control; that is, the test results were negative, indicating that AKK ONE had no mutagenic effect.

### 3.7. Bacterial Reverse Mutation Test (Ames Test)

The results of the bacterial reverse mutation test are shown in Table 6. Studies have shown that Dixon, sodium azide, methyl methylsulfonate, and 2-aminofluorene all significantly increased the number of reversed colonies, indicating that the experimental conditions are reliable. However, no cytotoxicity of AKK ONE was observed under the tested conditions. Regardless of the presence or absence of metabolic activation, the average numbers of reversed colonies counted in the AKK ONE treated group (5000 µg/plate) did not differ significantly from the control. These results showed that AKK ONE had no mutagenic activity against the strains tested, indicating that this strain did not cause gene mutation through base-pair changes or frame shifts in the bacterial genome under the conditions of this study. Our findings support the positive safety profile of AKK ONE.

### 3.8. Ninety-Day Oral Toxicity Study

The general behavior of animals is a key indicator to test the toxicity of drugs. Our results showed that AKK ONE did not cause any mortality or treatment-related clinical symptoms throughout the treatment period. No difference in food consumption or weight gain was found between the groups. All of the results did not exceed the normal range of Kunming mice of this age. Hematological values and biochemical indicators are important parameters to identify the toxicity of substances. There was no significant difference in hematological parameters, such as the white blood cell count, red blood cell count, hemoglobin, mean red blood cell volume, platelet count, lymphocytes, mean red blood cell hemoglobin concentration, and mean red blood cell hemoglobin, among all groups (Table 2).

The evaluation of liver and kidney function is also an important factor in determining the toxicity of a substance. Elevated levels of AST and ALT in the blood usually indicate hepatotoxicity [32]. AST and ALT levels in the cytoplasm above the limit are also signs of hepatotoxicity [33]. Creatinine, on the other hand, is a good predictor of kidney activity [34,35]. Therefore, the presence of abnormal amounts of these indicators in the serum can be used to identify any organ damage caused by toxicity. Table 3 shows that the intragastric administration of AKK ONE did not change the clinical biochemical parameters of mice.

The consumption of toxic compounds often affects the physiology and microstructure of organs and tissues. Table 4 shows that the weights of the organs were not affected by AKK ONE treatment. The histopathological analyses of the liver and kidney are illustrated in Figure 3c,d. Tissue micrographs revealed that the parenchymal structure of the liver and kidney in each group was regular in architecture. In this study, no signs of hyperemia or necrosis were observed. Compared with the saline control, there were no significant pathological differences in the color and texture of the liver and kidney tissues in the treatment group.

In the 90-day toxicity study, the no observed adverse-effect level (NOAEL) was 8.28 × 10^9^ cells /kg body weight/day, corresponding to a dose of 5.76 × 10^11^ bacteria in an average 70 kg person. These results indicated that AKK ONE is safe for long-term use at a dose of 8.28 × 10^9^ CFU/kg bw/day.

## 4. Conclusions

To summarize, we evaluated AKK ONE in terms of different aspects, including the origin, general characteristics, genes, tolerance to artificial gastrointestinal fluids, resistance to antibiotics, mutagenicity, and toxicity at the animal level. Overall, AKK ONE, an *A. muciniphila* strain isolated from the feces of healthy adults, was confirmed to have no virulence factors. AKK ONE is genetically stable and nonpathogenic to normal mice, thus further ensuring its safety as a probiotic candidate. Considering these findings together, we propose that AKK ONE is a safe *A. muciniphila* strain and exhibits great potential for use as a probiotic candidate.

## Figures and Tables

**Table 1:** Antibiotic resistance of AKK ONE to different antibiotics.

Antibiotics	Judging Rule			Tested Strain		Quality Control Strain		
				AKK ONE		*B. fragilis* ATCC25285		
	S	I	R	MIC (μg/mL)	Result	Quality Control Range	MIC (μg/mL)	Within the Quality Control Range?
Ampicillin	≤0.5	1	≥2	<0.25	Sensitivity	16–64	32	Yes
Ceftriaxone	≤16	32	≥64	8	Sensitivity	32–128	64	Yes
Cefotaxime	≤16	32	≥64	8	Sensitivity	8–32	32	Yes
Meropenem	≤4	8	≥16	0.125	Sensitivity	0.03–0.25	0.25	Yes
Tetracycline	≤4	8	≥16	2	Sensitivity	0.125–0.5	0.25	Yes
Moxifloxacin	≤2	4	≥8	128	Resistance	0.125–0.5	0.5	Yes
Chloramphenicol	≤8	16	≥32	4	Sensitivity	2–8	4	Yes

**Table 2:** Summary of hematological values in acute and subchronic toxicity studies.

Groups	Blood Routine Indexes	Saline	Low Dosage	Medium Dosage	High Dosage
Acute toxicity	WBC (×10^9^/L)	0.5 ± 0.3	0.6 ± 0.3	0.7 ± 0.7	0.6 ± 0.4
	RBC (×10^12^/L)	1.3 ± 0.7	1.7 ± 0.5	1.4 ± 0.5	1.20 ± 0.3
	HGB (g/L)	85.8 ± 25.3	105.5 ± 7.7	88.5 ± 16.2	88.5 ± 3.5
	MCV (fL)	54.7 ± 4.2	51.1 ± 4.1	51.2 ± 7.7	54.5 ± 1.3
	PLT (×10^9^/L)	1128.8 ± 488.3	1262.5 ± 351.4	975.5 ± 265.1	1374.5 ± 115.2
	Lymphocytes (×10^9^/L)	0.6 ± 0.2	0.6 ± 0.2	0.6 ± 0.6	0.6 ± 0.4
	MCHC (g/L)	1667.5 ± 1033.1	1288.0 ± 421.4	2435.1 ± 1506.1	1410.2 ± 434.1
	MCH (pg)	88.9 ± 47.5	86.8 ± 27.1	91.3 ± 56.2	76.9 ± 22.5
Subchronic toxicity	WBC (×10^9^/L)	1.8 ± 1.2	1.8 ± 0.1	1.6 ± 0.7	1.8 ± 0.1
	RBC (×10^12^/L)	7.4 ± 0.5	6.4 ± 0.4	7.7 ± 0.2	7.2 ± 0.6
	HGB (g/L)	107.5 ± 6.3	98.0 ± 6.6	108.5 ± 5.4	104.5 ± 8.1
	MCV (fL)	50.9 ± 2.5	50.1 ± 1.3	49.0 ± 1.5	49.8 ± 2.1
	PLT (×10^9^/L)	493.1 ± 57.3	474.7 ± 48.5	508.7 ± 63.0	473.0 ± 56.1
	Lymphocytes (×10^9^/L)	1.7 ± 1.0	1.8 ± 0.1	1.5 ± 0.6	1.8 ± 0.9
	MCHC (g/L)	286.7 ± 11.6	287.7 ± 8.9	287.2 ± 13.2	290.3 ± 12.3
	MCH (pg)	14.4 ± 0.6	14.4 ± 0.5	14.1 ± 0.6	14.4 ± 0.3

Abbreviations: WBC, white blood cell count; RBC, red blood cell count; HGB, hemoglobin; MCV, mean corpuscular volume; PLT, platelet count; MCHC, mean corpuscular hemoglobin concentration; MCH, mean corpuscular hemoglobin. Statistically significantly different from vehicle controls (*p* < 0.05).

**Table 3:** Summary of biochemical indicators in the serum and liver in acute and subchronic toxicity studies.

Groups	Indicators	Saline		Low Dosage		Medium Dosage		High Dosage	
		Serum	Liver	Serum	Liver	Serum	Liver	Serum	Liver
Acute toxicity	BG (mmol/L)	8.4 ± 1.7	9.1 ± 2.2	8.9 ± 1.6	8.9 ± 1.7	8.4 ± 1.5	9.4 ± 1.8	8.6 ± 1.8	9.1 ± 1.9
	TGs (mmol/L)	1.5 ± 0.8	0.4 ± 0.07	1.5 ± 0.7	0.4 ± 0.12	1.4 ± 0.6	0.4 ± 0.04	1.6 ± 0.7	0.4 ± 0.05
	TC (mmol/L)	5.9 ± 2.1	0.03 ± 0.01	6.4 ± 2.8	0.03 ± 0.01	6.6 ± 1.6	0.03 ± 0.01	6.4 ± 1.4	0.03 ± 0.01
	BA (μmol/L)	3.0 ± 0.8	56.2 ± 35.7	3.0 ± 0.6	50.1 ± 28.9	2.7 ± 0.7	52.4 ± 28.4	2.8 ± 0.4	56.6 ± 38.0
	TP (μg/mL)	261.4 ± 24.6	775.5 ± 74.0	289.6 ± 22.8	755.3 ± 80.0	267.7 ± 25.7	769.2 ± 70.6	285.1 ± 35.2	772.4 ± 69.2
	ALT (U/L)	15.3 ± 6.5	9.1 ± 4.7	18.8 ± 5.5	9.4 ± 3.7	15.9 ± 6.2	9.8 ± 1.7	16.8± 6.4	9.3 ± 3.7
	AST (U/L)	46.9 ± 9.1	10.1 ± 2.1	49.9 ± 8.4	8.9 ± 2.9	49.1 ± 5.1	9.4 ± 2.5	49.7 ± 4.8	9.0 ± 2.4
	Cr (μmol/L)	17.5 ± 5.1	-	23.1 ± 7.6	-	24.7 ± 8.8	-	18.8 ± 6.6	-
	BUN (mmol/L)	5.3 ± 1.7	-	5.0 ± 1.9	-	5.2 ± 1.7	-	5.1 ± 1.5	-
Subchronic toxicity	BG (mmol/L)	8.4 ± 1.5	12.4 ± 0.9	7.9 ± 1.3	12.6 ± 1.6	8.5 ± 1.6	11.8 ± 1.7	8.3 ± 1.2	11.7 ± 1.6
	TGs (mmol/L)	1.1 ± 0.5	1.8 ± 0.8	1.2 ± 0.3	2.0 ± 0.7	1.0 ± 0.3	2.0 ± 0.8	1.3 ± 0.6	2.1 ± 0.9
	TC (mmol/L)	2.0 ± 0.8	0.2 ± 0.1	2.0 ± 0.3	0.2 ± 0.1	2.0 ± 0.8	0.2 ± 0.1	2.3 ± 0.7	0.2 ± 0.1
	BA (μmol/L)	5.3 ± 1.7	2.7 ± 1.4	5.1 ± 2.1	2.1 ± 1.1	5.6 ± 2.5	2.5 ± 1.9	5.7 ± 2.5	2.3 ± 1.4
	TP (μg/mL)	1516 ± 363.7	621.7 ± 110.3	1484 ± 306.8	672.2± 121.7	1456 ± 303.2	639.1 ± 85.1	1573 ± 272.8	622.7 ± 74.2
	ALT (U/L)	10.1 ± 1.9	9.6 ± 2.7	11.3 ± 1.6	9.2 ± 2.2	12.3 ± 1.1	9.4 ± 2.6	11.9 ± 1.9	9.7 ± 2.6
	AST (U/L)	48.5 ± 6.2	278.2 ± 27.8	47.4 ± 9.0	267.3 ± 26.9	51.9 ± 5.1	272.2 ± 33.8	45.9 ± 8.8	280.4 ± 30.9
	Cr (μmol/L)	13.9 ± 3.9	-	12.7 ± 2.3	-	12.8 ± 5.8	-	14.5 ± 3.4	-
	BUN (mmol/L)	3.7 ± 0.7	-	3.8 ± 0.9	-	3.4 ± 0.4	-	3.4 ± 0.6	-

Abbreviations: BG, blood glucose; TGs, triglycerides; TC, total cholesterol; BA, bile acid; TP, total protein; ALT, alanine aminotransferase; AST, aspartate aminotransferase; Cr, creatinine; BUN, blood urea nitrogen; -, untested.

**Table 4:** Summary of relative organ weights (g/kg bw) in acute and subchronic toxicity studies.

		Heart	Liver	Spleen	Kidney	Thymus	Brain	Testicle	lung	Stomach	Intestines
Acute toxicity	Saline	5.8 ± 1.1	43.9 ± 7.5	2.5 ± 0.4	13.6 ± 2.5	2.9 ± 0.6	10.5± 1.5	6.2 ± 0.4	6.1 ± 0.9	19.3 ± 6.7	100.5 ± 10.6
	Low dosage	5.5 ± 1.1	46.8 ± 5.8	2.7± 0.9	14.0 ± 2.6	2.8 ± 0.5	11.2± 0.8	5.8 ± 0.3	5.7 ± 0.5	19.0 ± 2.2	101.2 ± 4.4
	Medium dosage	5.7 ± 0.9	47.4 ± 7.7	2.6 ± 0.3	14.6 ± 3.1	2.7 ± 0.3	9.9 ± 0.5	6.5 ± 0.6	6.4 ± 0.7	21.3 ± 5.4	97.9 ± 5.7
	High dosage	5.6 ± 1.5	49.9 ± 5.0	2.1 ± 0.8	13.4 ± 3.3	3.0 ± 0.2	10.6 ± 1.8	5.9± 0.6	5.9± 0.7	18.8 ± 5.5	100.4 ± 10.7
Subchronic toxicity	Saline	4.7 ± 0.4	39.6± 4.3	2.4 ± 0.7	11.2± 1.6	2.6 ± 0.8	9.4 ± 3.5	5.8 ± 1.1	5.8± 0.4	14.8 ± 5.9	77.4 ± 10.3
	Low dosage	4.7 ± 0.8	42.3 ± 5.0	2.7 ± 0.4	11.7± 0.9	2.8 ± 0.4	9.9 ± 1.6	5.9 ± 0.6	5.6 ± 0.5	14.6 ± 3.5	74.7 ± 9.7
	Medium dosage	4.6 ± 0.4	39.9± 6.4	2.5 ± 0.9	10.1 ± 3.9	2.5 ± 0.5	8.9 ± 2.2	5.5 ± 0.6	5.4 ± 0.5	15.3 ± 4.9	79.3 ± 14.4
	High dosage	4.6 ± 0.5	38.9 ± 3.2	2.7 ± 0.7	11.5± 1.0	2.7 ± 0.7	9.2 ± 1.9	5.3 ± 0.4	5.6 ± 0.6	14.4 ± 5.1	75.5 ± 14.8

**Table 5:** In vitro mammalian cell micronucleus test with AKK ONE.

Group	PCE (%)	NCE Ratio	PCE Ratio
Negative control group	36.34 ± 4.63	0.06 ± 0.09	0.09 ± 0.04
Low dose group	39.73 ± 5.62	0.02 ± 0.09	0.12 ± 0.04
Mid dose group	32.54 ± 5.79	0.03 ± 0.06	0.10 ± 0.09
High dose group	31.75 ± 6.74	0.03 ± 0.02	0.14 ± 0.03
Positive control group	24.6 ± 5.90	0.33 ± 0.10 ***	4.76 ± 2.13 ***

*** *p* < 0.001.

**Table 6:** Results of bacterial reverse mutation (Ames) test of AKK ONE.

Concentrations	Revertant Colonies per Plate (Mean ± Standard Deviation)									
	*Salmonella typhimurium*								*Escherichia coli*	
	TA97a		TA98		TA100		TA1535		WP2 *uvr A*	
	−S9	+S9	−S9	+S9	−S9	+S9	−S9	+S9	−S9	+S9
Distilled water control	122.00 ± 30.68	126.20 ± 22.13	41.40 ± 8.11	35.00 ± 6.40	144.60 ± 25.52	109.00 ± 43.81	12.00 ± 4.42	16.00 ± 6.52	122.40 ± 19.36	162.00 ± 32.41
*AKK* ONE	85.60 ± 14.54	99.20 ± 19.87	38.20 ± 12.09	34.00 ± 4.80	71.20 ± 9.58	62.00 ± 11.81	13.20 ± 4.49	13.00 ± 3.39	113.00 ± 13.91	134.20 ± 27.10
Dixon	1169.40 ± 181.47	-	1525.00 ± 271.69	-	-	-	-	-	-	-
Sodium azide	-	-	-	-	1347.00 ± 202.27	-	-	-	613.40 ± 61.99	-
Methyl methylsulfonate	-	-	-	-	-	-	141.80 ± 26.63	-	-	-
2-aminofluorene	-	1288.40 ± 296.29	-	1398.60 ± 309.13	-	1247.40 ± 59.71	-	163.20 ± 9.98	-	548.60 ± 111.81

Data are expressed as reversible colonies for each plate. Values are means ± SD (*n* = 3).

## Data Availability

The original contributions presented in the study are included in the article/Supplementary Materials; further inquiries can be directed to the corresponding author.

## Supplementary Materials

The following supporting information can be downloaded at https://www.mdpi.com/article/10.3390/foods13131979/s1, Figure S1: Morphological characteristic of AKK ONE; Table S1: Genome sequence characteristics of AKK ONE; Table S2: The proportion of genes to the genome annotated in NR, COG, GO, SwissProt, and KEGG; Table S3: Antibiotic-resistance genes analysis based on CARD database; Table S4: Antibiotic-resistance genes analysis based on ResFinder database; Table S5: Virulence factor analysis using VFDB database; Table S6: Prediction of pathogenicity of AKK ONE; Table S7: Biofilm formation ability of AKK ONE.
