# Supplementary material for: Genome- and Toxicology-Based Safety Assessment of Probiotic Akkermansia muciniphila ONE Isolated from Humans
Source: Foods. 2024 Jun 24;13(13):1979. doi: 10.3390/foods13131979 (PMC11241434; doi:10.3390/foods13131979)
Supplement: Supplementary file 1 [file foods-13-01979-s001.zip › foods-3026749-supplementary.pdf]

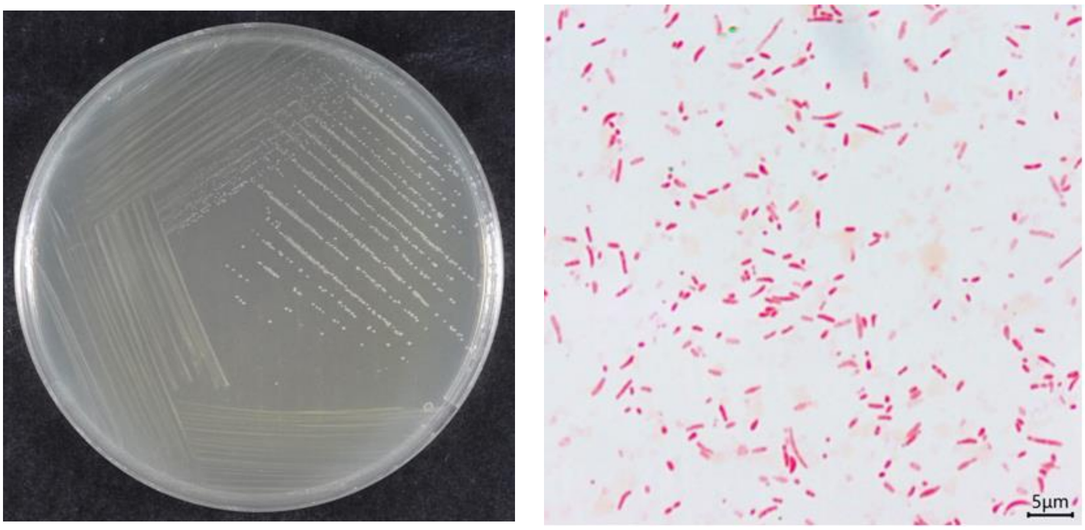

(**a**) (**b**)

Figure S1. Morphological Characteristic of AKK ONE. (a) Colony characteristics of AKK ONE in BHI medium. (b) Microscopic morphology of AKK ONE after Gram staining.

Table S1. Genome sequence characteristics of AKK ONE.

Sequence ID	Linear/Circular	Sequence Length	GC Content	A Content	T Content	G Content	C Content	N Content
chr	Circular	2,817,524	55.48%	631,592 (22.42%)	622,816 (22.11%)	775,880 (27.54%)	787,236 (27.94%)	0 (0.00%)
Total	•	2,817,524	55.48%	631,592 (22.42%)	622,816 (22.11%)	775,880 (27.54%)	787,236 (27.94%)	0 (0.00%)

Table S2. The proportion of genes to the genome annotated in NR, COG, GO, SwissProt and KEGG.

Database	Annotated_number	Unannotated_number
NR	2411 (83.22%)	486(16.78%)
SwissProt	1402 (48.39%)	1495(51.61%)
COG	1745 (60.23%)	1152(39.77%)
KEGG	1168 (40.32%)	1729(59.68%)
GO	1131 (39.04%)	1766(60.96%)

Table S3. Antibiotic resistance genes analysis based on CARD database.

ORF_ID	Cut_Off	Best_Hit_Bitscore	Best_Hit_ARO	Best_Identities	ARO	Drug Class	Resistance Mechanism	Percentage Length of Reference Sequence
Scaffolds_6_181376_115	Strict	763.8	*adeF*	41.55	3000777	fluoroquinolone antibiotic; tetracycline antibiotic	antibiotic efflux	99.72
Scaffolds_12_99539_36	Perfect	1254.6	*tetW*	100	3000194	tetracycline antibiotic	antibiotic target protection	100
Scaffolds_21_4974_3	Strict	598.2	*ANT(3'')-IIa*	100	3004089	aminoglycoside antibiotic	antibiotic inactivation	93.19

Table S4. Antibiotic resistance genes analysis based on ResFinder database.

Resistance gene	Identity	Contig or Depth	Position in contig	Phenotype	Accession
tet(W)	100.0	Scaffolds_12_99 539_36	1..1920	Doxycycline, tetra- cycline, minocy- cline	AJ427422
aadA1	99.75	Scaffolds_21_49 74_3	115..906	Spectinomycin, streptomycin	JQ414041

Table S5. Virulence factor analysis using VFDB database**.**

qseqid	sseqid	pident	e-value	bitscore	description
Scaffolds_11_100116_10	VFG046465(gb|YP _169203.1)	72.8	4.00E-177	615.1	(*tufA*) elongation factor Tu [EF-Tu (VF0460)] [*Francisella tularensis* subsp. *tularensis* SCHU S4]

Table S6. Prediction of pathogenicity of AKK ONE.

Probability of being a hu- man pathogen	In put proteome cov- erage (%)	Matched pathpgenic families	Matched not pathpgenic families
0.327	3.47	0	82

Table S7. Biofilm formation ability of AKK ONE**.**

Glucose	0%	0.25%	1%	2.5%
AKK.one	weak	weak	weak	weak

The experiments were repeated for *three times.* Values are means ± SD (n = 3).
